# Conversion of open tibial IIIb to IIIa fractures using intentional temporary deformation and the Taylor Spatial Frame

**DOI:** 10.1007/s11751-013-0160-0

**Published:** 2013-04-21

**Authors:** H. Sharma, T. Nunn

**Affiliations:** Hull Royal Infirmary, Anlaby Rd, Hull, HU3 2JZ UK

**Keywords:** Open fracture, Wound closure, Taylor Spatial Frame, Deformity correction

## Abstract

The closure of small-to-moderate-sized soft tissue defects in open tibial fractures can be successfully achieved with acute bony shortening. In some instances, it may be possible to close soft tissue envelope defects by preserving length and intentionally creating a deformity of the limb. As the soft tissue is now able to close, this manoeuvre converts an open IIIb to IIIa fracture. This obviates the need for soft tissue reconstructive procedures such as flaps and grafts, which have the potential to cause donor-site morbidity and may fail. In this article, the authors demonstrate the technique for treating anterior medial soft tissue defects by deforming the bone at the fracture site, permitting temporary malalignment and closure of the wound. After healing of the envelope, the malalignment is gradually corrected with the use of the Taylor Spatial Frame. We present two such cases and discuss the technical indications and challenges of managing such cases.

## Introduction

Gustilo and Anderson type III open tibial fractures are limb-threatening injuries with an average union time of between 28 and 52 weeks [1, 2]. Infection rates vary between reports and range from 6 to 50 % [3–5]. Considerable progress has been made in the treatment for these injuries, and agreed standards of care have been recently published [6]. The BOA and BAPRAS recognise that these injuries require joint care by orthopaedic and plastic limb reconstructive surgeons. Early joint management at a senior level is recommended in performing adequate wound debridement and planning further treatment.

Gustilo Mendoza and Williams subclassified type III fractures into A, B and C [7]. Type A had adequate soft tissue cover, and type B had inadequate and required a flap (local or free) to cover the exposed bone. Type C is associated with a vascular injury that requires reconstruction. This simple classification system is ubiquitously used despite its problems with interobserver reliability [8] and the wide spectrum of injury that falls into the IIIb category.

Reconstructive flaps for type IIIb fractures whether free or local do have donor-site morbidity and are not possible in every circumstance due to injury, patient and local vascular factors. Provision of a flap increases hospital stay and cost [9]. In some circumstances, it is possible to close the soft tissue defect once the limb is acutely shortened. This technique is well established and has good reported outcomes [10, 11]. The bony injuries that lend themselves to this treatment are circumferential bone loss where good bony contact can be re-established with acute shortening.

In cases where there is asymmetric cortical loss, closure of the wound may still be possible by performing an intentional temporary deformation of the tibia. Techniques of limb reconstruction mean that complex deformities can be accurately and gradually corrected. In this article, we describe two cases where angulation was intended to allow tension-free soft tissue closure, thus ‘converting’ a IIIb to IIIa fracture. The tibial deformities were corrected once there was certainty that the soft tissue envelope was well healed. This can be done with an Ilizarov frame but often requires complex frame configurations. Our cases were simply performed using the Taylor Spatial Frame ™(TSF), Smith & Nephew, Memphis, TN. The TSF, which is particularly useful for this technique, is a development from the Ilizarov frame that allows simultaneous correction of length, angulation, translation and rotation about a virtual axis. We believe that this method simplifies deformity correction and easily allows further corrections if the position is suboptimal.

## Case 1

**Fig. 1:**
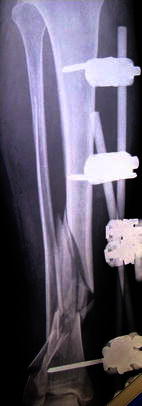
Open fracture distal tibia post-stabilisation with a bridging external fixator

**Fig. 2:**
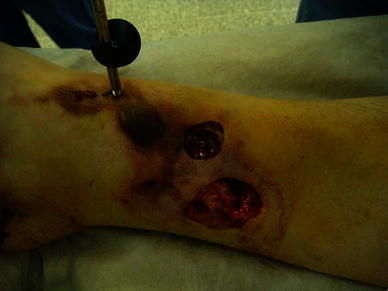
Two wounds, the smaller one communicated directly with the fracture site

**Fig. 3:**
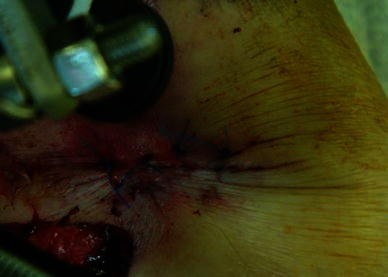
Mattress sutures to close the smaller wound that communicated with the fracture

**Fig. 4:**
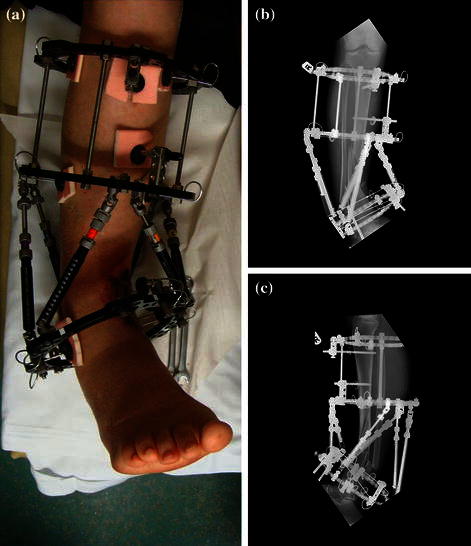
Waiting for the soft tissue envelope to heal. The intentional varus and recurvatum are seen with the TSF in situ. **a** Clinical picture. **b**, **c** AP & Lateral view of the intentional deformity

**Fig. 5:**
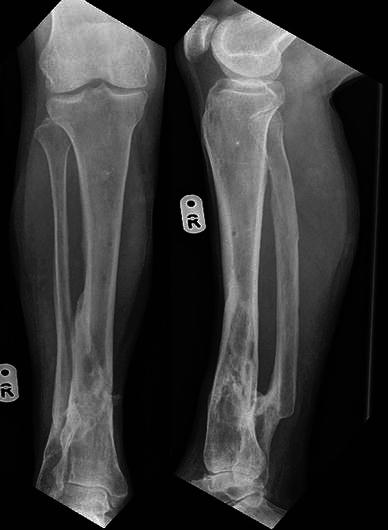
Radiographic position following solid union

A 60-year-old lady was thrown from her horse, sustaining an isolated IIIb open distal tibial fracture. Broad-spectrum antibiotics were commenced and the limb splinted in a backslab. She was fit and healthy and was doing well following her ipsilateral hip resurfacing procedure 3 years previously. The wound was debrided at 11 h post-injury, and the fracture stabilised with a temporary ankle-spanning external fixator (Fig. 1). The patient was transferred to our institution, and a second look was performed on day 3. Plans were made for definitive fixation and soft tissue envelope coverage achieved by using intentional deformation. There were two anteromedial wounds. Viable fracture fragments were exposed in the depths of the smaller wound. A simple dressing was placed over the two wounds, which measured 2 and 4 cm in diameter (Fig. 2). On day 10, the wounds were found to be clean. The external fixator was removed, and the smaller medial wound with exposed bone at the base was closed without significant tension by substantially deforming the leg through the fracture into 37° varus and 16° recurvatum. The other wound was left alone (Fig. 3). A Taylor Spatial Frame (rings-first technique) was used to stabilise the fracture. Antibiotics were discontinued the following day, and dressing changes were used to treat the second wound. Sutures were removed at 2 weeks (Fig. 4). The limb was kept in the intended deformed position for 6 weeks. Correction was commenced and took 4 weeks to complete. The patient required only occasional analgesics, and the consolidation of the fracture took a further 9 weeks until the frame was removed, 19 weeks after application (Fig. 5). During the consolidation phase, she had two broken wires, one of which was replaced in theatre 13 weeks after application.

**Fig. 6:**
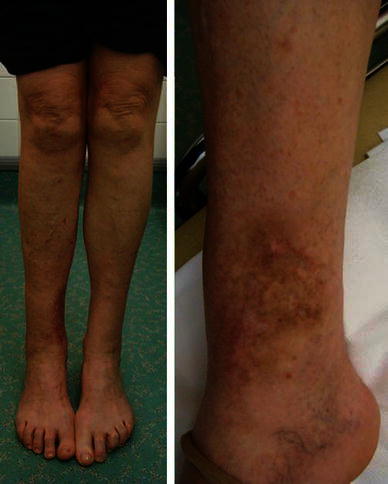
Clinical picture 1 year following injury

She was seen 1 year after injury (Fig. 6). She had received one course of antibiotics for suspected ‘infection’ which resolved. She was seen 2 years following injury without any problems and was discharged from our follow-up with a range of ankle and subtalar movements the same as the contralateral side. She scored maximal points on the EQ-5D summary index and visual analogue scale, the Iowa knee score and the Olerud and Molander activity score.

## Case 2

**Fig. 7:**
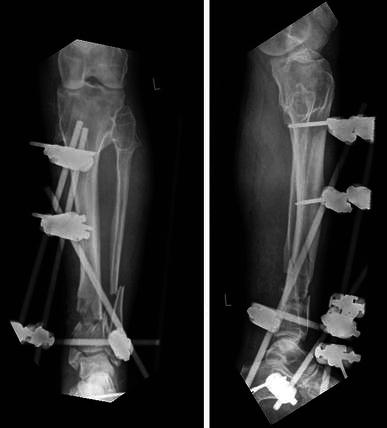
Temporary skeletal stabilisation of an open tibial fracture extending into the ankle joint. Note the typical features of multiple exostoses

**Fig. 8:**
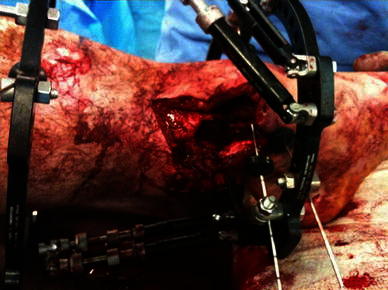
Large medial wound prior to deformation

**Fig. 9:**
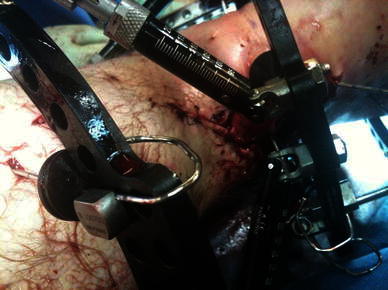
Medial wound closure, with intentional varus and recurvatum deformity present

**Fig. 10:**
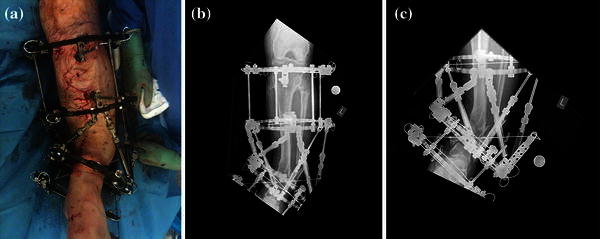
Frame with intentional deformity as seen from the front. **a** Clinical picture. **b**, **c** AP views showing intentional deformity

**Fig. 11:**
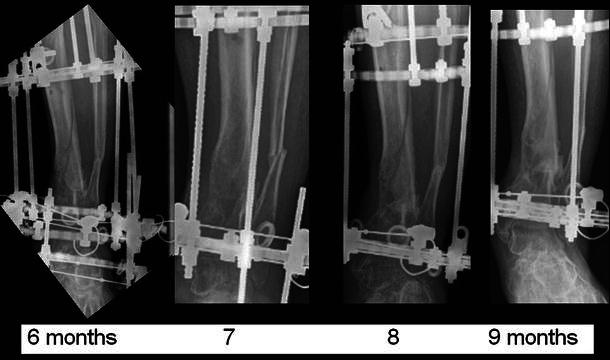
Sequential AP radiographs of the distal tibia with the frame in situ showing progressive fracture consolidation

**Fig. 12:**
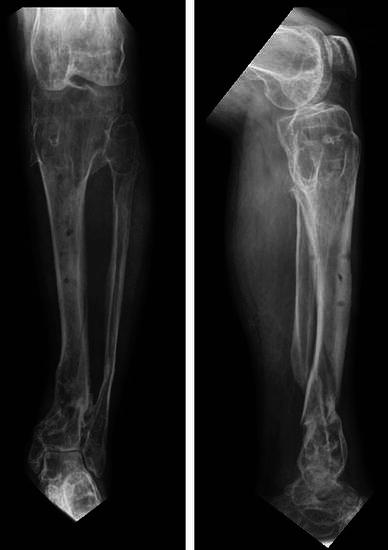
Radiographic position following solid union

A 48-year-old male pedestrian was hit by a car and sustained an isolated IIIb open fracture of his tibia. His medical history included multiple hereditary exostoses and alveolar microlithiasis, for which he had previously undergone a partial pneumonectomy. The patient was administered antibiotics and taken to theatre at 11 h post-injury. The wound was debrided and temporarily stabilised with an external fixator (Fig. 7). A CT of the extremity was obtained. The soft tissue defect was medial, measuring 8 cm by 4 cm. On day 3, an acute angulation at the fracture site was performed and the fracture stabilised with a Taylor Spatial Frame (Fig. 8). The wound was primarily closed without tension (Fig. 9), and the leg was held in intentional varus by 35° (Fig. 10). The wound was healed by 2 weeks and sutures removed. At 6 weeks, the correction was started and required only occasional morphine analgesics. Correction was completed at 11 weeks and, once achieved, the hindfoot element was removed from the frame and the fast-fix struts were substituted for rods. Four courses of oral antibiotics were required for pin-site infections during the treatment. At 9 months, the fracture gap had consolidated sufficiently for frame removal (Fig. 11). The patient had no leg problems following this and radiographs were satisfactory (Fig. 12).

**Fig. 13:**
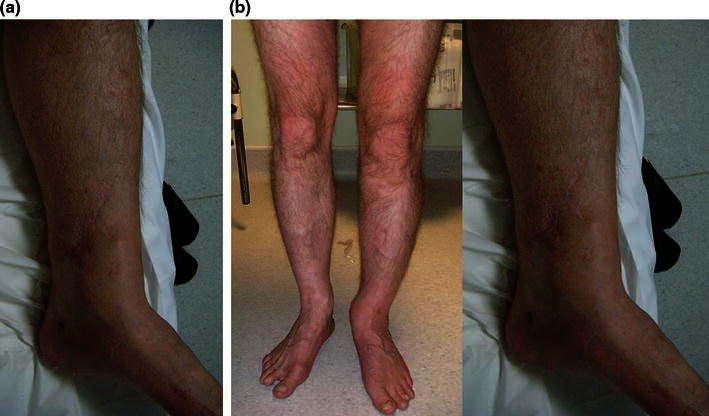
Clinical picture 18 months following injury

Eighteen months following the injury (Fig. 13), he had not returned to his previous physically active job principally due to breathlessness on exertion. This significantly affected his general wellbeing and is reflected in his EQ-5D visual analogue score of 70/100. His ankle was not painful, allowed him to walk, drive and climb stairs using a reciprocating gait. He complained of a degree of ankle stiffness. The range of ankle movement lacked 8° of dorsiflexion compared to the contralateral side, and the subtalar joint had a similar range of motion to the contralateral side. His Iowa knee score was maximal and the Olerud and Molander activity score was 85/100. Overall, he was satisfied with the outcome and would recommend the treatment.

## Discussion

In the management of grade IIIb fractures, fix and flap technique remains the standard of care that other techniques must be judged [3]. We reserve this technique for specific circumstances as evidence for its use as published in the English language literature is limited and often used in combination with acute shortening [12–16]. In fact, we are aware of only one previous case described in the English literature of the use of this technique without significant shortening. Gulsen and Özkan [17] used an Ilizarov frame and traditional hinge for a fracture that was 2 months old. Direct closure of a lateral wound was achieved after debridement with a 45° fracture-site angulation into a valgus position [17]. Gradual correction was commenced at 2 weeks. Stable union in 5° of valgus was achieved at 30 weeks from frame application.

Patient and injury selection for this technique is critical. Such decisions must be made by a surgeon proficient in the techniques of adult tibial deformity correction and be made in conjunction with the patient. In addition, backup options of a free flap and secondary reconstruction or amputation should be discussed with the patient in case the wound does not heal.

A number of factors should be borne in mind when selecting an appropriate patient.

### Injury factors

*Soft tissues*: Primary closure with temporary deformity is ideally suitable for IIIb open fractures with an anteromedial wound. This may be of variable size which should be possible to close without compromising vascularity. Adequate subcutaneous tissue must be present to support the skin and prevent wound breakdown during deformity correction.

*Fracture pattern*: A prerequisite of this technique is that bones should be deformable in the direction of the skin defect. This makes a fibula fracture close to the level of the tibial injury a necessity. The tibial fracture site may permit deformity if there is substantial comminution at the fracture site or bone loss on the medial side. We believe that fracture comminution is preferable and makes the deformation easy (this was found in both the illustrated cases presented). Distraction through a single fracture site inevitably means greater strain during correction. With multiple sites, the strain during the correction is shared [18]. We did not bone graft the cases described, and both had substantial comminution. Case 2 did have a small cortical defect. We did anticipate the need for a graft, but the gap filled in satisfactorily. All fragments of bone must be viable, and any devitalised fragments of bone should be removed at the primary debridement.

In both cases, correction was commenced late so as to ensure complete wound healing. We believe that it is not necessary to wait for 6 weeks provided the soft tissues are adequately healed. In a subsequent successful case, we were able to commence correction at 3 weeks. In delaying correction, we used distraction osteogenesis with manipulation of immature fracture callus at the fracture sites. The advantages of delaying the correction from 3 to 6 weeks may result in faster fracture union.

In the absence of comminution, overlapping the fracture fragments may be possible to allow shortening and wound closure, but we have no experience of this technique.

We believe that this technique may be suitable for open supramalleolar distal tibial fractures, open diaphyseal fractures and open fractures of the subtuberosity proximal tibia. As local flap options are often more restricted in the distal region and the deformity needed easier to gain, the distal tibial open fracture is most suitable for this technique.

### Patient factors

Careful counselling concerning the treatment plan, expectations from the patient and the likelihood of success should be discussed. In addition to usual circular frame issues, the deformity, which may be obvious for a prolonged period, must be anticipated and accepted by the patient. As mentioned, the timing of correction should be scheduled primarily according to soft tissue considerations. Compliance is essential and a good rapport maintained between patient and treating staff.

This technique may in fact be the only option for patients who are unsuitable for a local or a free flap due to chronic vascular problems. However, the patient should be informed of the need for amputation if it were to fail due to wound breakdown. Other scenarios where this technique may be of use are in situations where microvascular skills are not available such as in many developing countries. This may provide the orthopaedic trauma surgeon with deformity correction skills a potential solution. It is, however, a method that requires very close patient supervision and regular appraisal of progress.

### The technique

The TSF in our cases was applied using a rings-first method by passing wires orthogonal to each segment first. After adequate, minimal stabilisation of both bony segments, the tibia was deformed and soft tissue assessed for primary closure. When it was clear that soft tissue closure was possible, TSF application was completed. Strut removal on the side of skin defect and skin closure without tension was then performed. The TSF struts were then reapplied, and fixation was assessed for adequacy. Distal pulses and perfusion were always checked to ensure there were no secondary dysvascular consequences of the deformity. This may be anticipated as a result of severe kinking of vessels. The ankle was also assessed and the frame extended to the hindfoot if there was any concern regarding the adequacy of distal fixation. Frame extension may also be needed in the case of a lax floppy ankle due to deformity and relative tendon over-lengthening. The bridging element could be removed once the correction was performed to reduce stiffness in the ankle and hindfoot joints.

If, however, skin closure was not possible, then Ilizarov frame would have been applied and threaded rods adjusted to provide space for plastic surgeons to work for a local or free flap.

In the cases described above, we used the TSF software in total residual correction mode to achieve the deformity correction. We are aware of another method that uses direct scheduling (Lahoti O, personal communication). Here, the frame is applied noting the strut lengths both at deformation and once anatomically aligned. The struts are then gradually altered to achieve the desired anatomical alignment. This method does not require post-operative radiographic estimation or measurement of deformity parameters and is potentially easier.

### Post-operative treatment

Immediately following acute deformation, we remained alert for compartment syndrome and perfusion problems in the extremity. Elevation to reduce swelling is necessary. Prophylactic heparin was given to our patients. In the cases presented, we allowed partial weight bearing through the frame immediately, but weight bearing is dictated by the fracture pattern, the amount of deformity and frame configuration. The wounds were left to heal. If good bony contact was obtained at the site of bone loss, then deformed leg was left for up to 6 weeks. A slow correction was then performed to correct the deformity to allow the soft tissue envelope to stretch. If there was no bone loss, then deformed bone can be corrected early once skin is well healed (usually after 2–4 weeks). If there is any doubt about skin healing, then correction can be delayed as appropriate as in the cases presented. In the presence of bone loss, the rate of correction would be guided by the quality and quantity of callus formation and distraction adjusted accordingly. A delayed start to the correction may be beneficial as previously discussed. Any residual bone gap can be grafted later if callus is not evident. This should be ideally performed avoiding the initial traumatic wound and the surrounding skin.

## Conclusion

We presented two cases where acute deformation of the fractured tibia allowed primary skin closure and avoided the need for soft tissue flaps. We believe that this is a useful technique to consider in certain patients at centres where sufficient expertise with the TSF is available. The fracture must also be deformable in the direction of the wound to permit this. Further studies of this technique are necessary to improve our understanding of patient selection and outcomes.
